# The Pathology of Ague and the Agent That Produces It

**Published:** 1878-04

**Authors:** 


### Selections.

---

THE PATHOLOGY OF AGUE, AND THE AGENT THAT PRODUCES IT.—There is no subject, perhaps, in the long catalogue of diseases, that has attracted more attention of the medical profession than that of the pathology of what is known as miasmatic, or malarial fever. In 1695, Dr. Sancisi, a distinguished Italian physician, I believe, was the first to put forth distinct ideas and theories concerning the so-called malaria; and from that time to the present, new theories have been promulgated, soon to explode, leaving us as much in the dark as centuries ago, especially concerning the morbific agent that goes to produce the disease. Occasionally an over sanguine chemist has conceived the idea that he had discovered the peculiar poison known as miasm, and had possessed the erratic enemy in a tangible form; but he, too, has been forced to admit that the wily agent had eluded his grasp.

The profession has, for ages, with one accord, attributed all periodic fevers to the effect of miasma, a specific poison, absorbed and taken in the circulation of the blood, thereby chemically changing and contaminating it, a theory I have never been able to reconcile with the character of the disease. For the sake of convenience I shall use the terms miasma and malaria, but I believe there is another and better term for this agent, more consonent with the disease it produces, which I shall endeavor to explain as clearly as possible, and which I shall denominate the morbific electrical fluid.

In the first place, I cannot admit, and must deny, that malarial fever is a blood disease; and will submit as a fact demonstrable, that the blood is simply the vehicle which conveys the morbific agent, and no effect is produced until the structure for which the fluid has the greatest affinity has become ready, from some predisposing cause, to receive the impression of the agent, and thus will be specifically affected; which structure is the nervous system. The advocates of the chemical hypothesis contend that the constituents of the blood become altered and contaminated by the peculiar miasm or virus, but when such blood is introduced in the circulation of a healthy individual, it gives rise to nothing like the original disease.

Why it is that certain morbific agents select particular organs and tissues to exert their actions upon, we do not know; but that such is the fact all medical observers will bear witness. Neither is it more surprising than that some of the natural fluids of the body, like the urine, bile, etc., remain with impunity in some parts of the body, while if they gain admission to other parts, as the cellular substance, or peritoneum, they occasion inflammation, sloughing and death. So far, therefore, as determining by chemical analysis, the exact constituents of the morbific agent called malaria, may be regarded a delusive hope; as Liebig, in his animal chemistry, truly observes, "with all our discoveries we shall never know what light, electricity and magnetism are, in their essence. We can ascertain, however, the laws which regulate their motion and rest, because these are manifested in phenomena."

This is equally true, when applied to malaria, as I regard it, an electrical agent, produced in certain localities, possessing certain active principles which are latent and unappreciable in the natural state, and are only called forth and developed by the influence of some other agent or process which effects a transformation, or metamorphosis of the crude material. Thus it is with heat, electricity and magnetism; each only becomes apparent when certain physical substances operate upon each other in such a manner as to disturb or derange the original state of cohesion of particles. The morbific agent called malaria is produced in certain localities, by a combination of circumstances and elements, in certain definite proportions, and has an elective affinity for the nervous system; conveyed thence, *perhaps*, through the circulation of the blood, and if so, without impressing the great arterial system.

Any medical man who has had any experience in the treatment of ague must have been struck with the peculiar shock made upon the nervous system; an impression not unlike that of an ordinary electrical shock, only of longer duration and of milder form.

This electrical agent is not the result of decaying animal or vegetable matter, as is generally taken as a fact, but *may* be, and frequently *is*, generated in localities where there is no vegetation, either decaying or otherwise, and that are perfectly dry, and facts can be adduced to show that the decomposition of vegetable substances is only an accidental acccompaniment of the miasm, and not by any means an essential condition of its evolution.

Dr. William Furguson, an English physician of wide experience, in an interesting paper, "On the Nature and History of the Marsh Poisons," published in the Edinburgh "Philosophical Transactions," says, "In August, 1794, after a very hot and dry summer, our army in Holland encamped at Rosendaal and Oosterhout. The soil in both places was a level plain of sand, with a perfectly dry surface, where no vegetation existed or could exist, but stunted heath plants. It was universally percolated to within a few inches of the surface with water, which, so far from being putrid, was perfectly palatable. Here fevers of the intermittent and remittent types appeared among the troops in great abundance." Many instances of a similar character could be given. A striking example occurred under my own observation, a few years ago, in Baltimore city, Maryland. The northwestern part of the city is built on very high ground, and from the natural drainage is kept perfectly dry, and entirely free from either stagnant water or decomposing vegetable substances. Up to that time that part of the city was sparsely built, and owing to the healthfulness of the locality, a demand was created for residences, and the greater part of the unoccupied ground was built upon in the course of one or two years, in consequence of which a great deal of fresh earth was turned up, by excavating cellars, etc. Immediately thereafter intermittent fever set in, and prevailed for one or two seasons, scarcely a family escaping the disease; while prior to that time a case of ague was not known to orginate in that locality. The latter is another convincing proof, to my mind, of the fallacy of the theory of vegetable decomposition; but there was an element arose or brought into action, from or by the upturned earth, combining with another element necessary in the premesis, or being acted upon dynamically, which produced the morbific electrical agent causing ague. This electrical agent is rapidly produced, and one or more principles may exist naturally in all conditions, and the moment in which the substance of the atoms combines with the new element necessary to the production of the morbific agent, that moment it acquires the capacity of penetrating the organism, and exciting therein its deleterious effects. This agent brought in contact with the invisible extremities of nerves, its hyper-microscopic atoms will enter the organism at the same time with their superficial electricity, and will, if the nerves be in a perfectly natural state, be thrown out of the system without impediment, even after having penetrated it every direction. But if a body is in a state of imperfect health the power of conduction proper to the nervous substance will be materially diminished, and the morbific electric current leaves the atoms at the enfeebled spots, where they exert a detrimental influence. This electrical miasm is incapable of imparting its peculiar influence, unless it is brought into contact with those tissues for which it possesses a kind of elective affinity.

I am not sure that it is necessary for the miasma to be absorbed, and pass through the arterial system, to produce its effect on the human economy, but it may operate by making its primary impression upon the sentient extremities of the nerves, impairing their integrity, and rendering them incapable of conducting the electrical stimulus to the extreme vessels, and therefore produces the shock. The extreme terminations of the nerves are so highly impressible that the very minutest quantity of a specific agent is capable of producing prompt and decided effects, while the same agent would prove powerless if applied to the larger nerves. Thus it is that imponderable substances and mental emotions are so often the causes of disease. Sometimes a morbific agent may be powerless to exercise an influence upon any tissue, and may remain in the system an indefinite length of time without affecting it, and yet retain its activity. The reason for this may be explained thus: the tissues upon which it acts by affinity are in so perfect a state of vigor as to be able to resist the power of the noxious agent, until some cause shall enfeeble the part to be affected, and thus predispose it to receive the injurious impression.

The same explanation will justly apply to the latent principle of malaria, as often experienced by those exposed to its influence; the morbific agent remains harmless for months, when suddenly some tissue becomes enfeebled and incapable of resisting the action of the specific agent, and the disease in all its violence bursts forth. The nervous system is not unlike an electrical battery, possessing, as it, does, a peculiar imponderable, invisible principle, which may be thrown into active operation by coming in contact with another electrical element, producing good or evil effects, as to the agent employed.

The great nervous system may be properly likened unto an electrical battery; the various branches act the part of the poles for conveying the current; and when all the ramifications of the nerves are charged by this morbific electrical fluid, a physical impression is made upon the human economy, very like that of a shock received from an electrical battery.

In the effort of the noxious electrical fluid to expand itself upon the nervous system, the primary impression is that of rigor and shrinkage of parts, with contractility; the secondary impression is that of heat, caused by the rapid expansion of the parts previously acted upon, necessarily causing the evolution of heat, and what is known as the febrile stage of the disease.

As I said before, it is not necessary to have decaying animal or vegetable matter to produce this subtle agent, but it seems to require heat and moisture, and not the earthly substance of any particular locality; for instance, a subject predisposed to ague may eat heartily of water-melons, afterward expose himself to the heat of the sun for an hour or more, and he is a fortunate individual if he escapes a chill. Such instances of the rapid production of chills are numerous, and, certainly, we cannot attribute such attacks to the malaria of a marshy district; but it is a convincing proof that this subtle agent may be generated and produced in a very short period of time, similar to that of an electrical current, and the watermelon in such cases is simply a factor, the effect which, when brought in contact with agents natural or unnatural to the human system, subjected to the heat of the sun, generates the morbific electrical fluid, which, as soon as the battery (the nerve centres) is charged sufficiently, electrifies the whole nervous system, with all the pathognomonic symptoms of regular ague.

This agent possesses certain peculiar and distinct properties which enable it to exercise an influence only on particular nerves, and will pass over, in fulfillment of this law, various intermediate nerves of more direct anatomical connections. This principle of elective affinity, being so universal, as applied to morbific, as well as remedial agents, the influence which any substance of either class exerts upon the organic elements can, with propriety, be denominated its specific effect.

Therefore, this miasmatic electrical substance possesses the property of selecting that tissue for which it has an affinity, and of expending its entire primary action upon the particular parts selected; the sympathetic modifications, the result of variable degrees of strength and purity, operate mainly as secondary phenomena. It is by virtue of this law that medical men have been enabled to classify diseases; and that medicines may be administered which operate with certainty upon particular tissues and organs. The electrical influence of miasma is not entirely a new idea, as some writers long since attributed the operation of medicines to the same influence. Bischoff says: "All bodies, by contact with each other, act as electrics, without, however, necessarily undergoing any chemical changes.

Therefore, when a medicine is applied to the organism its action is electrical." The instantaneous effect of very minute quantities of hydrocyanic acid and some of the gases most certainly bear a close resemblance to the overwhelming shock of lightning, and therefore, it must be conceded, go far to sustain this opinion.

Whether all morbific and remedial agents act dynamically or electrically, may admit of a question, but it is quite clear to my mind that miasma is an electrical agent, and its primary impression is made upon some portion of the nervous system, and that its action is electrical. An individual may have but one chill, or a few, and no more; which may be explained in this wise: the amount of morbific agent generated in the system is dependent upon circumstances, just as is the case with the preparation of an electric battery. There is more than one agent necessary to the production of this agent, and there may be a deficiency of one or both in the human battery, and consequently only one or more charges are generated, after which there can be no more until the battery is supplied with additional elements. The time required for these elements to produce the morbific agent may be variable, according to the degree and quantity, and this may explain the periodic character of the paroxysms. Many theories have been advanced by pathologists, to account for the periodic occurrence of the paroxysms at regular and stated times; but reasoning upon the theory that miasma was a poisonous chemical agent, absorbed and taken in the circulation of the blood, producing a chemical change, thereby vitiating that fluid, of course, they found themselves at sea, and just when they thought they had grasped the miasmatic problem, their attempts at explanation have been either quite hypothetical, or totally insufficient and illogical.

One writer has ascribed the intermission to a periodic development of the fermentable matter in the blood. There has been no such development in the blood shown, nor any evidence of it; and if such were the fact, that such development took place, the question would recur, why such development should occur periodically.

Another referred the periodic character to some general law of the universe, in which he conceived some vague idea of such influences as the alternation of light to darkness, the ebbing and flowing of the tides, the periodic character of the seasons, etc:; but in this there is no rational explanation at all.

Still another advocates a most singular theory upon this subject. He attributes the periodic phenomena to the alterations necessarily produced in the human economy, especially in the functions of the circulation of the blood, by changing the position from the upright to the recumbent, and vice versa, every twenty-four hours, in the waking and sleeping periods. If such a theory was correct, it would not be necessary to resort to medication, but change the order of things, and refrain from the horizontal position during sleep, for a period. There have been many other and equally illogical and irrational theories promulgated in explanation of this peculiar phenomenon, but all have failed to satisfactorily enlighten the profession.

I am clearly of the opinion that the pathological theory of the periodic phenomenon can be accounted for, logically and rationally, when we have convinced ourselves of the nature of the agent that produces it; and I have endeavored to show, what is clear to my mind, that all the symptoms and phenomena of ague are attributable to an electrical morbific agent. This agent exists as an element of its own, perhaps, and only requires the action of some other power or substance to set it in motion, the same as producing the electric battery, which, when charged by the action of acids on certain metals, gives off the subtle fluid which produces the shock. This fluid exists in certain localities and under certain conditions. As before said, when there is a surface capable of absorbing moisture, having been flooded or soaked with water, then by a high temperature becomes dry quickly. This agent is received into a portion of the greater nervous system for which it has an affinity, and when the organic battery becomes so far charged with the fluid, it is conveyed off through the ramifications of nerves to the sentient extremities, until the battery has expended the charge. If there still remains a portion of the fluid in this organic battery, it immediately commences to increase in volume until the battery is again charged to its capacity, and again discharged, requiring greater or longer periods of time, according to the conditions of the battery, and the amount of the element in the battery.

The whole charge may be expended with one paroxysm, thus leaving the individual free from another attack until exposed to the same element that produced the original one. This organic battery may retain the fluid in a moderate degree for some considerable time, until there is some exciting cause to put it in motion and increase the charge. So subtle is this agent, that no evil effect or deleterious impression is made upon the system by its existence until the charge is sufficient to expend its force on the nervous extremities, producing thereby contraction of the tissues and the corresponding expansion, evolving heat and producing fever. As to the *methodus medendi*, I shall have but little to say, as my object has been mainly to inquire into the pathology of the disease and the morbific agent that produces it. One general principle, advocated by Paine in his "Institutes of Medicine," I shall quote as consonant with my own views as to the treatment. He says:

"All diseases consist in a modification of the vital properties, and a consequent change of function, and are therefore only a variation of the natural states; the artificial cure consists in a restoration of those properties and functions, by making upon the former certain impressions which enable them to obey their natural tendencies to a state of health.

"Remedial agents of positive virtues operate like the truly morbific, but less profoundly in their therapeutical doses, and the philosophy of their cure consists in establishing in a direct manner certain morbid alterations in the already diseased properties and actions of life, which are more conducive to the natural tendency that exists in the vital properties to return from a morbid to their natural states."

While cinchona and its salts are improperly regarded specifics for the cure of ague, they no doubt possess in a wide degree the requisite properties for arresting the production of the morbific electrical fluid; but salicylic acid, although used by me to a limited extent, comparatively, fulfilled the ends more satisfactorily than any drug in my hands. The *modus operandi* of the cure of ague is not by the change of tissue produced by the remedy used, by being absorbed into the circulation of the blood. The remedy, to effect a cure, need not be such as will produce any change of tissue, but such as has a negative electrical character—one that will neutralize or negative the organic battery, and thus destroy the charge and prevent an accumulation of the fluid in the human battery; in short, one that has an affinity for the nervous system.

I have purposely refrained from suggesting, more than incidentally, any particular remedy or plan of treatment in this paper, but confined myself to the pathology of the disease, hoping that future research may develop into use a remedy more reliable and better suited to the malady under consideration. It occurs to me, however, that electrical or tetanic remedies are indicated, as we have abundant proof of the action of sudden and electrical impressions in arresting an attack of ague by such means as joy and fright, and more pointedly, by the subject being suddenly and unexpectedly plunged into water. I am aware that some of my suggestions savor somewhat of the Hahnemannic doctrine, but I claim that we, as a profession, are strictly eclectic, and everything in pathology, physiology and therapeutics belongs to us as such, and it is not only our privilege, but our duty, to seek that which is good and profit by it.

The views set forth in this paper are not entirely of recent origin, as my note-book shows that I promulgated similar theories as to the pathology of ague as far back as 1859, in private intercourse with medical friends, and many agreed with me as to the nervous connections.

I know that any theory promulgated that is so entirely at variance with the preconceived views of the profession will meet with criticism, perhaps severe; but if conducted with kind and generous intentions, can only result in good; and such I heartily seek, as I hold my views subject to correction, and possibly to refutation.—*Dr. A. S. Stonebraker in Philadelphia Medical and Surgical Reporter.*

